# Pathogenic and Protective Autoantibodies in Autoimmune Polyendocrinopathy-Candidiasis-Ectodermal Dystrophy (APECED)

**DOI:** 10.3390/antib6010001

**Published:** 2017-01-17

**Authors:** Sakeen W. Kashem, Bryce A. Binstadt

**Affiliations:** Center for Immunology, Department of Pediatrics, Medical School, University of Minnesota, Minneapolis, MN 55455, USA; kashe007@umn.edu

**Keywords:** APECED, autoantibodies, T cells, AIRE

## Abstract

Autoimmune polyendocrinopathy-candidiasis-ectodermal dystrophy (APECED) is a rare disorder caused by mutations in the autoimmune regulator (*AIRE*) gene, leading to defects in T cell selection. The disease manifestations include both autoimmune tissue destruction and immunodeficiency, with specific susceptibility to chronic mucocutaneous candidiasis. Studies have demonstrated a wide repertoire of high affinity tissue- and cytokine-specific antibodies in patients with APECED. Here, we review the antigenic targets and function of these disease-causing and disease-ameliorating antibodies.

## 1. Introduction

Autoimmune polyendocrinopathy-candidiasis-ectodermal dystrophy (APECED; also referred to as autoimmune polyendocrine syndrome type 1 (APS1)) is a rare disorder caused by autosomal recessive mutations in the autoimmune regulator (*AIRE*) gene; a less severe disease phenotype has been associated with dominant negative mutations in the same gene [1,2,3]. Dysfunctional AIRE protein results in both primary immunodeficiency and tissue-specific autoimmunity. AIRE is a transcriptional factor that promotes the expression of many tissue-restricted self-antigens on medullary thymic epithelial cells (mTECs) to enforce central T cell tolerance via negative selection [4]. In addition, there is evidence that AIRE has a role in peripheral T cell tolerance [5]. Impaired T cell tolerance leads to the subsequent dysregulation of B cell somatic hypermutation with high affinity autoantibody production [6]. Thus, although *AIRE* mutations primarily lead to defects in T cell tolerance, the subsequent autoantibody production drives many of the disease manifestations in patients with APECED.

The incidence of APECED varies from approximately 1:9000 to 1:25:000 in Finnish, Sardinian and Iranian-Jewish populations to 1:200,000 in Western Europeans [7,8,9]. Clinically, APECED is defined by the presence of two of the three major disease components of chronic mucocutaneous candidiasis (CMC), primary adrenal insufficiency, and hypoparathyroidism. Notably, both CMC and the endocrinologic manifestations of APECED are associated with specific autoantibodies. In addition to polyendocrinopathies, multiple other organ systems have been demonstrated to be affected in patients’ APECED including autoimmune dermatologic, hepatic, renal and intestinal disease [10].

The presentation of APECED, including the autoantibody repertoire and distinct organ involvement, varies among patients. In fact, the clinical presentation can be highly variable even amongst siblings with identical *AIRE* genotypes, suggesting the contribution of other modifier genes and environmental factors [11]. This is further supported by studies in *Aire*-deficient mice that demonstrated differing patterns of tissue-specific autoantibodies depending upon the background mouse strain, suggesting that individual major histocompatibility complexes may influence the disease phenotype [12]. In this review, we detail the various tissue- and cytokine-specific autoantibodies described in patients with APECED, based on established and emerging literature. Table 1 summarizes the tissue-specific autoantibodies.

## 2. Antibodies against Tissue Antigens

### 2.1. Anti-Endocrine Antibodies

Patients with APECED suffer from tissue-specific autoimmunity affecting various organs. The most common tissues affected are those of the endocrine system including parathyroid, thyroid, adrenal, pituitary and gonadal organs. Amongst the endocrine disorders, hypoparathyroidism and adrenal insufficiency are prevalent in >80% of APECED patients.

Hypoparathyroidism generally appears within the first decade of life and in a recent U.S. study was the most frequent, and often the only, endocrine disease [10]. Interestingly, the majority of Iranian Jews, but only 20% of Finnish patients, present with hypoparathyroidism as the only endocrinopathy [39]. One possible parathyroid autoantigen that has been identified is NACHT, leucine-rich repeat (LRR), pyrin domain (PYD) leucine-rich-repeat protein 5 (NLRP5) [13]. NLRP5 is a cytoplasmic receptor of unknown significance that is expressed most specifically in the chief cells of the parathyroid gland and in oocytes. NLRP5-specific autoantibodies were detected in almost half of APECED patients with hypoparathyroidism but were absent in all APECED patients without hypoparathyroidism [11]. Furthermore, only 6% patients with idiopathic hypoparathyroidism and fewer than 3% of healthy controls had autoantibodies against NLRP5, suggesting that NLRP5 may be a tissue-specific autoantigen in APECED [40]. In addition, autoantibodies against the calcium-sensing receptor (CaSR) were detected in almost 40% of Finnish APECED patients, although this study questioned the specificity and sensitivity of anti-NLRP5 and CaSR antibodies as markers of hypoparathyroidism [41].

Adrenal insufficiency typically follows hypoparathyroidism in the disease course of APECED, with an age at onset of approximately 4–12 years. It presents with typical symptoms of Addison’s disease including fatigue, weight loss, hyperpigmentation, hypoglycemia and hypotension [10]. Many patients with APECED display autoantibodies against 21-hydroxylase (CYP450c21) years before the adrenal insufficiency becomes clinically apparent. In addition, 17α-hydroxylase (CYP450c17) and side-chain cleavage enzyme (CYP450scc) can also serve as autoantigens of the adrenal glands and are associated with autoimmune destruction of the adrenal zona glomerulosa and zona fascicualata, characterized by increased plasma levels of renin and/or adrenocorticotropin hormone along with low cortisol levels (reviewed in [14]). Antibodies to NLRP5 have also been associated with adrenal insufficiency [15]. Notably, some studies of *Aire*-deficient C57BL/6 mice demonstrate adrenal autoreactivity while others do not [42,43]. Thus, the mouse models of APECED do not possess full fidelity to the human disease.

Autoantibodies against NLRP5, CYP450c21, CYP450c17 and CYP450scc have also been associated with hypogonadism in APECED patients [13,15,17]. Gonadal failure occurs in approximately 38% of females and 21% of males with APECED in the U.S. [10]. Ovarian failure presents with autoantibodies against CYP450scc with symptoms of amenorrhea, and/or absent, slow, or regressing pubertal development, as well as elevated plasma concentrations of follicle-stimulating hormone or luteinizing hormone at baseline or after stimulation with gonadotropin-releasing hormone [8]. Male gonadal failure manifests as subfertility that presents around the fourth decade of life [10]. In addition to the autoantibodies against CYP molecules, autoantibodies against the prostate antigen transglutaminase 4 (TGM4) can be found in adult male patients with APECED [44]. TGM4 is a major regulator of semen viscosity and promotes semen coagulation [32]. *Aire*-deficient mice fail to express *Tgm4* mRNA in mTECs, produce high serum titers of anti-TGM4 autoantibodies, and develop generalized prostatic inflammation and male subfertility [44]. Recently, *Aire* was demonstrated to be expressed in the uterus and to play a key role in the deciduation and implantation of embryos [45]. Thus, Aire may play a role in fertility that extends beyond its role in thymic selection.

Type 1 diabetes (T1D) is a rare manifestation of APECED that occurs in approximately 1%–18% of patients [26]. Its prevalence is higher among Finnish patients than among patients of Middle Eastern, Eastern or Western European ancestry [39]. Given the variation of disease manifestation amongst different ethnic groups, it was initially hypothesized that various other factors such as human leukocyte antigen (HLA) risk alleles and environmental exposures predisposed Finnish patients to higher rates of T1D. Interestingly, while clinical T1D rarely occurs in APECED patients, high titers of antibodies against pancreatic autoantigens glutamic acid decarboxylase isoform 65 (GAD65) and tyrosine phosphatase-related islet antigen 2 (IA-2) were demonstrated in over 50% and 10% of U.S. patients, respectively [10]. In comparison, approximately 70%–80% and 32%–75% of newly diagnosed T1D patients *without* APECED have detectable circulating GAD65 and IA-2 autoantibodies, respectively [27]. Autoantibodies against IA-2 but not GAD65 were significantly associated with the time to development of diabetes in APECED patients [10]. Other less common pancreatic auto-antibodies found in Finnish APECED patients include islet cell, insulin and GAD67 antibodies [26]. Recently, it was reported that high-titer, neutralizing anti-IFN-α antibodies were present in APECED patients without T1D, whereas similar but non-neutralizing anti-IFN-α antibodies were present in APECED patients with T1D. This finding correlates with previous reports of type 1 interferons being pathogenic in T1D (discussed in greater detail below) [6].

Other endocrine organs affected in APECED include the thyroid and pituitary glands. Hypothyroidism occurs in approximately 22% of U.S. patients and generally presents in the third decade of life [10]. A dominant heterozygous missense *AIRE* mutation resulting in a G228W amino acid substitution was associated with a high prevalence of autoimmune thyroiditis in an Italian study of APECED patients [46]. The most common thyroid autoantibodies found in APECED are anti-thyroglobulin (TG) and -thyroid peroxidase (TPO) [10]. While anti-TG and anti-TPO antibodies are commonly found in patients with Hashimoto’s thyroiditis and cause thyroid follicle destruction, only approximately 10% of APECED patients demonstrate thyroid-specific antibodies [10]. It is unknown whether *Aire*-deficient mice have autoantibodies to thyroid antigens spontaneously but do produce such antibodies after immunization with antigens. Hypopituitarism is a rare manifestation of APECED and presents most commonly with isolated growth hormone (GH)-deficiency and less often with hypogonadotropic hypogonadism, central diabetes insipidus, adrenocorticotropic hormone deficiency or multiple pituitary insufficiencies. Growth hormone deficiency occurs in approximately 17% of U.S. patients and typically presents within the first decade of life [10]. In addition, autoantibodies against pituitary tudor domain containing protein 6 (TDRD6) has been demonstrated to be present in almost half of the patients with APECED but not in patients with T1D, systemic lupus erythematous, lymphocytic hypophysitis or other autoimmune diseases [30,31], but the TDRD6 autoantibody titers do not seem to correlate closely with hypopituitarism in APECED. TDRD6 has an unknown role in pituitary function. TDRD6 is reportedly required for spermiogenesis, and there is a greater correlation of anti-TDRD6 antibodies with gonadal failure in APECED [47]. Similarly, some patients develop antibodies directed against pituitary enolase and other cytosolic proteins, but these are not necessarily associated with clinical hypopituitarism, suggesting more generally that some “bystander” autoantibodies develop after tissue destruction and can be useful diagnostically, but are not the primary mediators of organ-specific pathology [48].

### 2.2. Anti-Cutaneous Antibodies

APECED patients can present with non-endocrine, organ-specific autoimmune conditions. Urticarial eruption was seen in almost two-thirds of APECED patients in an American cohort and was the most common initial manifestation of APECED with an onset most commonly occurring early in life [10]. While histological examination of skin biopsies of APECED patients with urticarial eruption demonstrates patchy mixed myeloid and lymphoid infiltrates, it is unknown whether autoantibodies play a role in the disease process [10]. Similarly, nail dystrophy or ectodermal dysplasia was found in 17% of APECED patients without a clear identification of an autoantigen [10]. Given that *AIRE* is expressed by keratinocytes, it may have specific regulatory function in skin tissue that may drive some of the cutaneous disease manifestations in APECED [49].

Vitiligo and alopecia were present in 37% and 17% of an American APECED cohort, respectively [10]. Alopecia typically presents generally in the first decade, while vitiligo occurs in the second. Autoantibodies against tyrosine hydroxylase, an enzyme found in hair follicles and the central nervous system, and against GAD65 may be associated with alopecia and vitiligo [36,37]. In addition, antibodies against SOX9/10 are increased in the sera of APECED patients with vitiligo but are rarely found in patients with isolated vitiligo [38]. However, it is unknown whether these antibodies are directly pathogenic or whether the cutaneous manifestations are caused primarily by autoreactive T cells.

### 2.3. Anti-Gastrointestinal Antibodies

Gastrointestinal diseases are prevalent in approximately 25% of U.S. patients with APECED [10]. They generally manifest in the second decade of life and include disorders such as autoimmune hepatitis, atrophic gastritis with or without pernicious anemia, and malabsorption. Thus, patients may present with symptoms of diarrhea, constipation, nutritional deficiencies and/or growth impairment.

One such autoimmune manifestation of APECED is atrophic gastritis. Autoimmune atrophic gastritis (AAG) is an autoimmune disease affecting the gastric parietal cells and intrinsic factor (IF) that leads to eventual gastric atrophy, vitamin B12 deficiency and pernicious anemia [18]. While not always symptomatic, biopsy-proven gastritis was seen in approximately half of American APECED patients [10]. APECED patients with AAG have antibodies against the sodium–potassium channel molecule of the parietal cells (anti-parietal cell antibodies) in the fundus of the stomach and, more commonly, against IF itself [8]. IF is a secreted glycoprotein produced by parietal cells that is needed for the absorption of vitamin B12 from the ileum. Low serum vitamin B12 is associated with the extent of anti-parietal cell and anti-IF antibodies and can manifest as atrophic glossitis, diarrhea, malabsorption, macrocytic anemia and pancytopenia [19]. Further reports of autoimmune gastritis in APECED include a case report of a patient with malabsorption and pernicious anemia with a loss of gastrointestinal endocrine cells (GIEC) associated with circulating anti-GIEC but not anti-parietal cell antibodies [20].

Certain strains of *Aire*-deficient mice develop gastritis. In addition, the transfer of serum from *Aire*-/- NOD or Balb/C, but not C57BL/6, mice into *Rag*-deficient hosts results in the deposition of antibodies in the stomach and other organs [50]. This suggests that other genetic factors play a role in addition to *Aire* in driving multisystem autoimmunity in APECED. Antibodies against mucin 6 of the stomach mucosa and protein disulfide isomerase-associated 2 of the stomach and pancreas were demonstrated in *Aire*-deficient mice of various strains [12]. Thus, autoantibody-driven gastritis is a frequent finding of APECED in both human patients and mouse models.

Intestinal dysfunction was also present in over 50% of North American APECED patients [10]. Tryptophan hydroxylase (TPH) was identified as an intestinal autoantigen, and autoantibodies against TPH were present in approximately 80% of American APECED patients [10,25]. Anti-TPH antibodies were present in over 90% of APECED patients with intestinal dysfunction but only in 30% of those without intestinal disease; such antibodies were not detected in patients with Crohn’s disease, celiac disease, or other autoimmune enteropathies [25]. TPH is expressed in serotonin-producing cells in the central nervous system and intestine [51]. TPH antibodies in APECED patients correlated with gastrointestinal symptoms and the absence of enterochromaffin cells of the mucosa and small bowel. In addition, autoantibodies against histidine decarboxylase of enterochromaffin cells have also been identified in APECED patients with intestinal dysfunction [52]. Some APECED patients also demonstrated α-defensin-specific antibodies and the loss of enteric Paneth cells [53]. Similarly, *Aire*-deficient mice developed α-defensin-specific autoantibodies and intestinal pathology [53].

### 2.4. Anti-Hepatic Antibodies

Autoimmune hepatitis (AIH) has been reported in approximately 10%–20% of U.S. patients with APECED during the first or second decades of life [10]. Anti-liver microsomal antibodies were detected in approximately 50% of APECED patients with hepatitis compared to only 11% of APECED patients without AIH. In *Aire*-deficient mice, approximately 20% of animals develop autoantibodies against various liver antigens to drive hepatitis [50].

Hepatitis in APECED patients is associated with antibodies directed against CYP450 antigens CYP1A1, CYP1A2, CYP2A6 and CYP2B6. Autoantibodies against CYP1A2 were found only in APECED patients with hepatitis whereas the presence of other autoantibodies was less specific [21]. In addition to liver microsomal antibodies, autoantibodies against aromatic-L-amino acid decarboxylase (AADC) expressed in liver-cytosol as well as in other tissues are significantly increased in APECED patients with hepatitis (92%) and vitiligo (82%) [22,23,24]. AADC is a key decarboxylase enzyme involved in the biosynthesis of major neural and peripheral neurotransmitters such as dopamine, serotonin, histamine and others. Anti-nuclear antibodies are common among patients with AIH, but are detectable in only 9% of APECED patients with AIH, similar to the prevalence of anti-nuclear antibodies in healthy controls [21].

More generally, only 25% of patients with APECED have detectable anti-nuclear antibodies, a feature of several more common autoimmune diseases; similarly, antibodies associated with other more common autoimmune diseases such as rheumatoid arthritis, celiac disease, and bullous pemphigoid are not commonly detected among patients with APECED [54]. This finding underscores the notion that tissue-restricted autoantibodies in patients with APECED arise via mechanisms distinct from those that drive the majority of autoimmune diseases.

### 2.5. Antibodies against Other Tissue-Restricted Antigens

Ocular manifestations occur in approximately 15%–25% of U.S. APECED patients, including keratoconjunctivitis that can present with keratopathy, dry eye, sublenticular cataracts, iridocyclitis, retinal detachment and optic atrophy [10]. Untreated autoimmune keratoconjunctivitis in APECED patients can lead to blindness [55]. In addition, approximately 40% of APECED patient’s develop Sjogren’s-like syndrome without the antibodies to extractable nuclear antigens (e.g., Ro, La) that typify idiopathic Sjogren’s syndrome [10]. While no autoantigens have been identified in APECED patients with keratoconjunctivitis or Sjogren’s-like syndrome, *Aire*-deficient mouse models have demonstrated autoantibodies against odorant binding protein 1a of lacrimal glands that lead to ocular dryness [16].

Tubulointersitial nephritis (TIN) is a rare but serious complication of APECED that can lead to moderate to severe renal failure [10]. It occurs in less than 10% of APECED patients. It can lead to chronic interstitial nephritis requiring renal transplantation. In one specific case, TIN recurred in the transplanted kidney and was successfully reversed with the therapeutic B cell-depleting monoclonal antibody, rituximab [56]. Thus, humoral responses can play a pathogenic role in driving renal disease in APECED. Immune complex deposition in the kidneys of APECED patients can appear as focal granular/linear IgG and C3 staining of tubular basement membranes and also mesangial depositions of C3 and C1q and IgM [56]. Further studies have found circulating anti-proximal tubular autoantibodies in all APECED patients with TIN [35].

Autoimmune interstitial lung disease manifests in approximately 40% of North American APECED patients with a mean onset of 10.5 years of age [10]. Autoantibodies against a putative potassium channel regulator (KCNRG) were found in the sera of approximately 28% of APECED patients with pneumonitits and 0% of APECED patients without pneumonitis [10]. Autoantibodies against KCNRG are not observed in patients with allergic asthma, chronic obstructive pulmonary disease, Sjogren’s syndrome, or other autoimmune pulmonary diseases [28]. KCNRG expression was restricted to the epithelial cells of terminal bronchioles. Interestingly, KCNRG autoantibodies were identified in an APECED patient with multiple hospital admissions for respiratory insufficiency and B cell lymphocytic aggregates on lung biopsy. Treatment of this patient with rituximab led to improvement in symptoms, suggesting a pathogenic role of humoral immunity in driving pneumonitis in APECED patients [33]. In addition to KCNRG, antibodies to bactericidal/permeability-increasing fold-containing B1 (BPIFB1) of the bronchial epithelium have been identified in 85.7% and 14.3% of APECED patients with and without pneumonitis, respectively [10]. Patients without APECED but with interstitial lung disease have also been demonstrated to harbor autoantibodies to BPIFB1 [34]. *Aire*-deficient mice may also develop pulmonary autoantibodies, including antibodies against BPIFB family of proteins, and develop lung disease depending on the background strain of the mice [34].

## 3. Anti-Cytokine Antibodies

### 3.1. Pathogenic Antibodies

In addition to tissue-restricted antibody responses, many high-titer, high-affinity antibodies to several cytokines have been described in nearly all APECED patients. Some of these anti-cytokine antibodies have shown to be pathogenic in causing chronic mucocutaneous candidiasis (CMC). CMC is one of the earliest signs of APECED, usually occurring within the first decade of life [10]. In studies of North American patients with disease, CMC was present in approximately 86% of patients. The most common sites of candidiasis include oral thrush, vulvovaginal candidiasis, esophageal candidiasis and nail/cutaneous candidiasis [10]. Given that untreated esophageal candidiasis increases the risk of squamous cell cancer, APECED patients demonstrate higher incidences of esophageal cancer [19]. There has been no association of APECED with fatal disseminated candidiasis, protection against which is mediated by a distinct immunological pathway from that responsible for immunity against CMC [57].

The occurrence of CMC suggested that APECED patients have a component of immunodeficiency in addition to their multi-system autoimmunity. CD4+ T cells (Th17) and innate immune cell secretion of IL-17 and IL-22 have been demonstrated to be imperative for effective immunity against *Candida albicans* in humans and mice [58]. Ineffective production or signaling of IL-17 due to genetic or acquired reasons is known to predispose to the development of CMC [58,59]. T cells from APECED patients with CMC demonstrated significantly reduced IL-17F and IL-22 production after stimulation with *C. albicans* antigens. Most importantly, the reduction of these responses was strongly correlated with the presence of neutralizing autoantibodies to IL-17F and IL-22 [60]. Antibodies against IL-17A, IL-17F, and IL-22 were prevalent in approximately 40%, 75% and 91% of APECED patients, respectively, in an initial study of >150 patients [60]. These anti-cytokine antibodies were infrequent in APECED patients without CMC [10]. Further studies have since reproduced a similar prevalence of anti-IL-17 and anti-IL-22 antibodies in other APECED cohorts [61,62]. Interestingly, anti-IL-17 and anti-IL-22 antibodies were also found in CMC patients with thymomas with aberrant expression of *AIRE*, suggesting that dysregulation of central tolerance drives the generation of anti-cytokine antibodies and subsequent CMC development [60]. In addition to IL-17 and IL-22 antibodies, APECED patients demonstrated autoantibodies specific for IL-6, whereas IL-23 autoantibodies were present in approximately 27.9% of thymoma patients but not in APECED patients [63]. Both IL-6 and IL-23 have been shown to be required for effective immunity against mucocutaneous *C. albicans* [58,64,65]. Antibodies against IL-1β, IL-21 or TGF-β were not found in either APECED or thymoma patients [63]. *Aire*-deficient C57BL/6 mice do not have increased susceptibility to oral or disseminated candidiasis, nor do they generate anti-IL-17 antibodies [42,66]. In contrast, aged *Aire*-deficient BALB/c mice have been shown to have neutralizing autoantibodies to IL-17A and IL-17F and to have increased susceptibility to Candidiasis [66]. Thus, it is possible that distinct genetic background and major histocompatibility complex (MHC) play a role in anti-cytokine antibody development and immunity against candidiasis in the mouse models of APECED.

### 3.2. Protective Antibodies

In addition to anti-IL-17 and -IL-22 antibodies, some APECED patients have detectable autoantibodies against IL-32γ and IL-20 and nearly all have high-titer autoantibodies to several type I IFNs [6]. The presence of anti-IFN antibodies among APECED patients was first reported a decade ago by Meager and colleagues [67]. The serum titers of anti-IFN antibodies can exceed 1:1,000,000, much greater than the titers of autoantibodies to tissue-restricted antigens [68]. Type I IFNs are secreted by stromal cells and leukocytes in response to engagement of toll-like receptors by viruses or by endogenous nucleic acids in the case of autoimmune diseases. While APECED patients have antibodies to unique sets of autoantigens, nearly all APECED patients have autoantibodies against IFN-α subtypes and to IFN-ω [10]. Furthermore, anti-cytokine antibodies from patients were of extreme high affinity and were biologically active in vitro and/or in vivo [6]. Notably, while nearly all patients with APECED have antibodies against IFN-α subtypes and IFN-ω, they do not have an increased susceptibility or incidence of viral infections [6]. This is unexpected, based on the increased susceptibility to severe viral infections among patients with deficiencies in downstream mediators of IFN signaling [69]. One possible reason for preserved anti-viral immunity in APECED patients with anti-IFNα antibodies may be due to lack of autoantibodies to IFN-β and IFN-γ, allowing these other cytokines to exert their anti-viral effects [6].

Interestingly, although some APECED patients do develop T1D with autoantibodies, other APECED patients appear to be protected from developing T1D. A recent study reported that APECED patients with neutralizing anti-IFN antibodies were *less* likely to have T1D than APECED patients without neutralizing anti-IFN antibodies [6]. This finding is consistent with reports that type 1 IFNs are pathogenic in mouse models of T1D [6]. Thus, it is hypothesized that these neutralizing anti-IFN antibodies protect APECED patients from developing T1D. Further studies will be needed to understand whether these neutralizing anti-type 1 IFN antibodies can dampen other type 1 IFN-driven diseases such as systemic lupus erythematosus.

Although extensive studies have not yet been performed, it is postulated that APECED patients may have a decreased incidence of psoriasis due to the presence of circulating anti-IL-17 and anti-IL-22 antibodies. Patient-derived autoantibodies against IL-22 reduced imiquimod-induced psoriasis-like inflammation in mice [6]. Thus, APECED patients may have disease-ameliorating anti-cytokine antibodies that inhibit the development of specific autoimmune diseases that otherwise might be expected to occur.

Why APECED patients make autoantibodies against soluble cytokines remains unclear. Some investigators have speculated that tissue destruction via autoimmune, viral or traumatic attack might lead to the release of tissue-specific autoantigens in an inflammatory milieu, which subsequently results in autoantibody formation against these proteins. However, it is important to note that anti-cytokine antibodies in APECED can appear sometimes as early as the neonatal period and CMC can develop in patients at an early age [10,70]. Furthermore, while patients demonstrate antibodies against certain cytokines, APECED patients do not have antibodies against IFNγ, IFNβ, GM-CSF, IL1α, IL1β or IL12p70 [10]. It seems unlikely that tissue damage would only result in antibody production against only specific interferons and Th17-associated cytokines, suggesting that other explanations are at play.

Studies in mice have suggested that *Aire* may have functional roles in addition to mediating thymic negative selection of T cells. For example, *Aire* is expressed by thymic B cells as well as extra-thymic cells [71,72]. It is therefore possible that anti-cytokine antibodies are generated due to defective peripheral expression of *Aire*. However, it should be noted that patients with thymomas can produce anti-IL-17 and anti-IFN antibodies, consistent with *AIRE*’s role in modulating central immune tolerance [60]. Given that cytokines are expressed and secreted in the thymus, it is also possible that *AIRE* deficiency may indirectly prompt the thymic environment to be more immunogenic than tolerogenic to those cytokine antigens [73,74].

Finally, it is also possible that anti-cytokine antibodies occur as a result of molecular mimicry. The loss of B cell tolerance during somatic hypermutation leads to anti-cytokine antibodies of very high affinities in APECED patients, likely due to the provision of help from autoreactive T cells that have not been negatively selected appropriately. Dysregulated somatic hypermutation may lead to the production of mutated, high-affinity self-reactive antibodies from B cells, perhaps initially primed by exogenous antigens. Such models of molecular mimicry to unrelated antigens have been proposed for the generation of IFNγ autoantibodies in patients with mycobacterial disease, thyrotropin receptor antibodies, pemphigus autoantibodies, and anti-GM-CSF autoantibodies in pulmonary alveolar proteinosis [75,76,77,78]. Patients with these conditions demonstrate a much more limited repertoire of autoantibody specificities than do APECED patients; in contrast, the broad T cell selection defects in patients with APECED might explain why they develop autoantibodies against such a wide array of tissue-specific antigens as well as cytokines.

## 4. Conclusions

Although the fundamental defect in patients with APECED is one of central T cell tolerance, most of the disease manifestations appear to be associated with or directly caused by autoantibodies. We have reviewed the current state of knowledge regarding pathogenic autoantibodies in patients with APECED, including antibodies directed against tissue antigens as well as cytokines. We have also reviewed emerging literature suggesting that some patients with APECED generate anti-cytokine antibodies that protect against the development of certain autoimmune diseases. Understanding the mechanisms leading to the generation of anti-cytokine autoantibodies in patients with APECED promises to reveal new insights into the basic mechanisms of immune tolerance.

## Figures and Tables

**Table 1:** Tissue-specific autoantibodies identified in patients with APECED (Autoimmune polyendocrinopathy-candidiasis-ectodermal dystrophy).

Tissue	Antigen	Disease Outcome	References
Adrenal, testis, ovary	CYP450c21	Addison’s disease or Gonadal failure	[10,13,14,15]
	CYP450c17		
	CYP450scc		
		NLRP5	
Eye	OBP1a	Ocular dryness	[16]
Gastric	Intrinsic factor	Malabsorption	[12,17,18,19,20]
	GIEC	Pernicious anemia	
	Mucin 6	Gastritis in mice	
Hepatic	CYP450 1A1	Hepatitis	[21,22,23,24]
	CYP450 1A2		
	CYP450 2A6		
	CYP450 2B6		
	AADC	Hepatitis/vitiligo	
Intestinal	TPH	Enteropathy	[10,25]
	α-defensin	Loss of Paneth cells	
Pancreas	GAD65	T1D	[26,27]
	IA-2		
	GAD67		
Parathyroid	NLRP5	Hypoparathyroidism	[28,29]
	CaSR		
Pituitary	GH	Hypopituitaritism	[10,30,31]
	TDRD6	Unknown Possible gonadal failure	
Prostate	TGM4	Infertility	[32]
Pulmonary	KCNRG	Interstitial lung disease	[33,34]
	BPIFB1		
Renal	proximal tubular	tubulointerstital nephritis	[35]
Skin	tyrosine hydroxylase	Alopecia areata/vitiligo	[36,37,38]
	GAD65		
	SOX9/10	Vitiligo	
Thyroid	TG	Hypothyroidism	[10]
	TPO		
